# A Longitudinal Study of Families Created Using Egg Donation: Family Functioning at Age 5

**DOI:** 10.1037/fam0001145

**Published:** 2023-09-14

**Authors:** Susan Imrie, Joanna Lysons, Sarah Foley, Vasanti Jadva, Kate Shaw, Jess Grimmel, Susan E. Golombok

**Affiliations:** 1 Centre for Family Research, University of Cambridge; 2 Moray House School of Education and Sport, University of Edinburgh; 3 Institute of Women’s Health, University College London

**Keywords:** assisted reproduction, egg donation, emotional availability, parent–child relationships, child adjustment

## Abstract

Findings are reported from Phase 2 of a longitudinal study of family functioning in heterosexual-couple families with 5 year olds conceived using identity-release egg donation. Seventy-two egg donation families were compared to 50 in vitro fertilization (IVF) families (ethnicity: 93% White British) using standardized observational, interview, and questionnaire measures. There were no differences between family types in the quality of mother–child or father–child interaction, apart from lower structuring by fathers in egg donation families. Egg donation mothers and fathers reported higher levels of parenting stress and lower levels of confidence and competence than their IVF counterparts. Egg donation mothers reported lower social support and couple relationship quality, greater anger toward their child, and perceived their child as more angry and less happy, compared to IVF mothers. Egg donation fathers showed greater criticism and anger toward their child, less joy in parenting, and were less satisfied with the support they received, than IVF fathers. Children in egg donation families showed higher levels of externalizing problems than IVF children as rated by mothers, fathers, and teachers, whereas they were rated as having higher levels of internalizing problems by teachers only. Externalizing problems were predicted by mothers’ lower initial social support, steeper increases in parenting stress and greater concurrent criticism, whereas internalizing problems were associated with poorer initial couple relationship quality as rated by mothers. Both were predicted by fewer gains in reflective functioning. There was a moderation effect such that parenting stress was a stronger predictor of externalizing problems for egg donation than IVF families.

Since the birth of the first baby born through in vitro fertilization (IVF) in 1978, more than 8 million children have been born worldwide using assisted reproduction (European Society of Human Reproduction and Embryology, 2018). An increasingly common type of assisted reproduction is IVF with donor eggs, which was developed for use by women who were unable to use their own eggs in their fertility treatment (Lutjen et al., 1984). In families created in this way, the child does not share a genetic relationship with their mother. Data on the number of live births from IVF cycles using donor eggs from 2018 show that they resulted in over 10,000 live births in the United States and over 1,200 in the United Kingdom, with numbers increasing yearly (Centers for Disease Control and Prevention, 2021; Human Fertilisation and Embryology Authority, 2020). Treatment using egg donation most commonly involves either anonymous donation, or identity-release donation, where the child may request the donor’s identity on reaching adulthood. Whereas most existing research on family functioning in egg donation families comprises samples, where parents have used anonymous donation to conceive, the present study constitutes the second phase of a longitudinal study of egg donation families with identity-release donors.

Concerns about families formed through egg donation have focused on the lack of a genetic relationship between the mother and the child. Evolutionary psychology theories, such as kin selection theory, argue that parents’ investment in their offspring is disproportionately biased toward those with whom they share genetic material, and that genetically unrelated children are negatively affected by withheld parental investments (Timming & French, 2021). However, a substantial body of research on new family forms has challenged these theories, showing that family processes matter more for children’s healthy psychological development than the composition of the family (Imrie & Golombok, 2020; Lamb, 2012). This body of work notwithstanding, families formed through egg donation still challenge powerful Western sociocultural norms that prioritize genetic over social relatedness in kin relationships (Freeman, 2014) and assume and privilege genetic relationships between mothers and their children (Park, 2013). Research with adoptive parents has found that mothers may perceive higher levels of stigma than fathers about nongenetic parenthood (Goldberg et al., 2011), suggesting that nongenetic motherhood may be particularly challenging.

Longitudinal data on egg donation families have come from two studies: the European Study of Assisted Reproduction Families (Golombok et al., 1999; Murray et al., 2006) and the U.K. Longitudinal Study of Assisted Reproduction Families (Golombok et al., 2004, 2005, 2006, 2011, 2013, 2017, 2023). The former compared outcomes for egg donation, sperm donation, adoptive, and IVF families at two time points, when children were aged 3*–*8 years and 12 years. The latter followed a sample of children born in 2000 over seven time points and compared family functioning in egg donation, sperm donation, and unassisted conception families. In both studies, children had been conceived using anonymous egg donation.

Findings from the larger of the two studies found a consistently high quality of parent–child relationship at ages 1, 2, and 3 years, with egg donation mothers and fathers showing more positive parent–child relationships than unassisted conception parents at age 1 (Golombok et al., 2004), egg donation mothers expressing greater joy in the relationship at age 2 (Golombok et al., 2005), and a higher quality of interaction with their child at age 3 (Golombok et al., 2006) than the two comparison groups. At age 7, however, gamete donation mothers’ interactions with their children were less positive than those of the unassisted conception group in an observational assessment, which appeared to be explained by parents’ disclosure of the child’s origins, with the lower scores driven by the nondisclosing parents (Golombok et al., 2011). At age 14, poorer relationship quality was similarly found between egg donation mothers and their children compared to sperm donation mother–child dyads on parental acceptance/rejection and family relationship problems as reported by questionnaire by both mothers and adolescents (Golombok et al., 2017). There were no group differences on observational or interview assessments of mother–child relationship quality. Again, more positive relationships were found between mothers and adolescents in families, where parents had disclosed the child’s origins by age 7. While this literature suggests that egg donation families seem to have good quality relationships overall, it does point to some subtle differences between families created through egg donation and other family forms. It is also worth noting that observational assessments were not included in this study before middle childhood.

In terms of child psychological adjustment, children conceived using egg donation were found to be well-adjusted in the European Study of Assisted Reproduction Families at both time points (Golombok et al., 1999; Murray et al., 2006), although the egg donation sample comprised only 21 families at Phase 1 and 17 families at Phase 2. The U.K. Longitudinal Study of Assisted Reproduction Families showed that children and adolescents showed good psychological adjustment at all phases of the study, with no differences between groups, as assessed using the widely used Strengths and Difficulties Questionnaire (SDQ) from age three onward (Golombok et al., 2005, 2006, 2011, 2013, 2017, 2023). A survey of more than 700 British parents with 5*–*9 year olds conceived through five types of assisted reproduction found higher levels of conduct problems in egg donation children than in children conceived through IVF and sperm donation as rated by fathers, although scores were in the normal range and no differences were found in mother-report data (Shelton et al., 2009). No differences were found in parents’ ratings of child anxiety, depression, peer problems, or prosocial behavior, with the latter two constructs also measured using the SDQ. The only study to have examined child adjustment in identity-release egg donation families involved 83 egg donation families and 113 donor insemination families with children aged 7*–*8 years in Sweden and found children’s scores to be within the normal range (Widbom et al., 2022).

Most research on egg donation families has included samples who used anonymous donation, whereas very little empirical work has been carried out with families who used identity-release donation. With increasing numbers of countries banning the use of anonymous donation on the grounds that it denies children important information about their biological heritage, and moving toward identity-release donation, this is a particularly pressing gap in the literature. Identity-release donation is not without its challenges. The prospect of the child learning the donor’s identity in the future and potentially being able to contact them may be threatening for some parents and offer less clear boundaries between the two families (Lysons et al., 2022, 2023). The process of deciding whether and how to disclose the child’s donor conception to them may also pose different challenges in identity-release families, as disclosure may have a different meaning to parents, given that the child may discover the donor’s identity in the future (Isaksson et al., 2016) which may add greater complexity to these decisions (Freeman et al., 2016).

The first phase of the present study, when the children were aged one, was the first examination of identity-release egg donation families (Imrie, Jadva, Fishel, & Golombok, 2019; Imrie, Jadva, & Golombok, 2019, 2020). One hundred and fifty two-parent, different-sex couples with infants were recruited through U.K. fertility clinics, and families formed through identity-release egg donation were compared to families who had used IVF with their own gametes to control for the use of fertility treatment.

Although findings from Phase 1 indicated that the families were functioning well overall, some significant differences between groups were identified that highlighted the importance of assessing family functioning in this particular family type longitudinally. With regard to the mother–infant relationship quality, an observational assessment using a free-play task coded using the Emotional Availability Scales (Biringen, 2008) found that egg donation mother–infant dyads showed less optimal interaction quality than IVF mother–infant dyads (Imrie, Jadva, Fishel, & Golombok, 2019). Specifically, egg donation mothers showed less sensitivity and less structuring with their infants, and their infants were less responsive to, and involving of, their mothers. These differences were of a medium effect size. Furthermore, the egg donation mothers had lower perceived social support than did IVF mothers, and egg donation fathers had poorer psychological health than IVF fathers (Imrie, Jadva, & Golombok, 2019). Moreover, qualitative analysis of interview data with mothers revealed that a minority struggled with the idea of nongenetic motherhood during their child’s infancy (Imrie et al., 2020). It is well-established within developmental science that factors including the quality of parent–child interaction, parental psychological well-being, and parental representations of the parent–child relationship are associated with child adjustment (Lamb, 2012; Luyten et al., 2020). With this in mind, the findings from Phase 1 indicated the importance of following the families up at a later stage, particularly to establish whether differences in mother–child relationship quality persisted, and whether these differences had any long-term effects on children’s psychological adjustment.

Phase 2 of the study examines family functioning when the children were 5 years old. In the United Kingdom, this is the age by which children will have started their transition to school, a key life cycle event that poses new social, emotional, and behavioral challenges (Pianta & Cox, 1999). Moreover, specific to this sample, many parents will have begun the process of telling their children about their donor conception by age 5. Fertility clinics in the United Kingdom generally advise patients that they should start telling their children about this from an early age (Human Fertilisation and Embryology Authority, 2021), and it has been shown that most families formed through anonymous egg donation have done so by age 5 years (Ilioi et al., 2017).

The first phase of the study, with its primary focus on relationship quality, was informed by attachment theory, which foregrounds the importance of the quality of the caregiver–infant relationship as the foundation for children’s later socioemotional development across multiple outcomes (Bowlby, 1982), including social competence (Groh et al., 2017), and internalizing and externalizing problems (Madigan et al., 2016). Both of the measures used to assess parent–infant relationship quality at Phase 1 were derived from attachment theory and, in combination, assessed the relationship at both the representational and behavioral level, which increases understanding of the organization of the relationship (Korja et al., 2010).

The current phase of the study is informed by attachment theory and a relational developmental systems approach, both of which focus on bidirectional relations between the social environment, parenting and child psychological adjustment (Osher et al., 2020; Overton, 2015). The empirical and theoretical literature on parenting indicates that negative aspects of parenting, such as criticism, hostility, conflict, and parental psychopathology are associated with more negative child adjustment, whereas more positive parenting constructs, such as sensitivity, warmth, cooperative coparenting, and parental psychological well-being are associated with more positive child outcomes. Correspondingly, child characteristics influence parental psychological well-being and the quality of parenting (Bornstein, 2019; Osher et al., 2020).

The current phase examined whether parents’ psychological well-being and the quality of parent–child relationships continued to differ between groups in early childhood. In line with the Phase 1 findings, and in view of the concern that mothers who lack a genetic connection to their children may feel less confident in their parental role, especially when faced with the prospect of their child discovering the identity of their egg donor in future, we predicted that mother–child relationship quality would be less optimal in egg donation families. Consistent with Phase 1 findings, we also predicted that egg donation parents would show poorer psychological well-being compared to IVF families, but that there would be no differences between family types in father–child relationship quality.

We also examined whether children born through identity-release egg donation were at risk for psychological problems in early childhood, and if so, to identify the nature of these problems and the mechanisms involved. In line with the theoretical framework and findings from the first phase of the study, we predicted that children in families created using egg donation would show higher levels of adjustment difficulties, and that differences would be associated with quality of parenting, including parental psychological well-being, quality of parent–child interaction and parental social support.

## Method

### Participants

The present study reports on a sample of 122 mothers (*M*_age_ = 45.43 years, *SD* = 4.93 years), 96 fathers (*M*_age_ = 47.12 years, *SD* = 6.53 years), and 122 children (56 female, 66 male, *M*_age_ = 5.63 years, *SD* = .32 years) who took part in the second phase of a longitudinal comparative study of families with children born through egg donation (*n* = 72) or IVF using their own gametes (*n* = 50). Of these families, 63 women conceived using identity-release donors and nine using known donors. Sixty-six percent of the children’s teachers also participated. The majority of mothers and fathers were highly educated (62.4% of mothers and 52.8% of fathers had an undergraduate or graduate degree) and most parents identified their ethnicity as “White British” (95.9% of mothers, 90.4% of fathers). As illustrated in Table 1, mothers were, on average, older in egg donation (*M*_age_ = 47.63, *SD* = 4.35) than IVF families (*M*_age_ = 42.23, *SD* = 3.92), *t*(120) = 6.94, *p* < .001, Cohen’s *d* = 1.28, as were fathers in egg donation (*M*_age_ = 48.48, *SD* = 6.48) compared with IVF families (*M*_age_ = 44.29, *SD* = 8.41), *t*(120) = 3.11, *p* < .001, Cohen’s *d* = 0.57. Fewer children in egg donation families had siblings than those in IVF families, χ^2^(1) = 11.67, *p* = .009, Cramér’s *V* = .31. Families did not differ in terms of other key demographic characteristics, including working status, education, perceived financial difficulties, and prior psychiatric contact (see Table 1). Three (4.2%) egg donation, and nine (18%) IVF parents had separated since Phase 1.[Table tbl1]

The sample at Phase 2 represents 81.3% of the families who participated in the initial phase, with 78.7% of fathers participating at Phase 2 (Imrie, Jadva, Fishel, & Golombok, 2019). Specifically, six families were unable to be traced and 22 families declined to take part. Excluding the families who could not be traced, the participation rate was 85.3%. Retention did not differ by family type, χ^2^(1) = 1.47, *p* = .225. The retained sample did not differ from those who did not participate in terms of key demographic variables (e.g., parent age, education qualification, income) or in the main variables of interest in the present study (i.e., Phase 1 parenting stress, couple relationship quality, social support, or reflective functioning).

### Procedure

Phase 2 of the study was conducted between April 2018 and December 2019. All of the researchers were highly experienced in collecting data from families created by assisted reproduction. Three interviewers (JL, KS, and JG) visited families in their homes for a 2–3 hr visit when their child was 5 years old. Written informed consent was obtained from each parent. As far as possible, parents completed a similar procedure to Phase 1 (Imrie, Jadva, Fishel, & Golombok, 2019). Each parent was administered an audio-recorded semistructured interview and completed a questionnaire booklet assessing psychological well-being, the quality of the relationship with their partner, social support, and the psychological adjustment of their child. Mothers and children, and fathers and children, were also filmed for 10 min completing a structured activity (Etch-a-Sketch or building blocks). To acknowledge their time, families received a small gift token and a small gift for their child. Written permission was also obtained from parents to contact the children’s teachers, who were asked to complete a questionnaire on the child’s adjustment. Written informed consent was obtained from teachers. The protocol was approved by the University of Cambridge Psychology Research Ethics Committee.

### Measures

#### Parental Psychological Well-Being

##### Parenting Stress

The short form of the Parenting Stress Index (PSI-SF; Abidin, 1990) was administered to parents to assess stress associated with parenting. The PSI-SF has 36 items. Total scores range from 36 to 180, with higher scores reflecting greater parenting stress. The short form correlates highly with the full-length version of the PSI, for which predictive and concurrent validity have been demonstrated (Abidin, 1990). Cronbach’s α for the present study were .94 (mothers) and .93 (fathers).

##### Social Support

Parents were administered the Multidimensional Scale of Perceived Social Support (Zimet et al., 1988). The questionnaire has 12 items which produce three subscale scores of parents’ perceived adequacy of support from family, friends, and a significant other. For each subscale, higher scores reflect higher perceived social support. The questionnaire has good test–retest reliability and validity (Zimet et al., 1988). Mean scale scores of 1*–*2.9, 3*–*5, and 5.1*–*7 are classified as low, moderate, and high support, respectively (Zimet et al., 1988). Cronbach’s α were .95 (mothers) and .92 (fathers).

##### Couple Relationship Quality

The 36-item Golombok Rust Inventory of Marital State (GRIMS; Rust et al., 1990) was administered to parents to assess the quality of the couple relationship. The total score ranges from 0 to 84, with a higher scores reflecting poorer relationship quality. Relationship dissatisfaction is indicated by a score of greater than 34. The GRIMS discriminates significantly between couples who are about to separate and those who are not (Rust et al., 1990). Cronbach’s α were .93 (mothers) and .93 (fathers).

#### Parent–Child Relationship Quality

Parent–child relationship quality was assessed using (a) an observational measure of parent–child interaction quality, (b) a representational measure of the parent–child relationship, and (c) an interview-based measure of criticism of the child.

##### Observational Measure of Parent–Child Interaction Quality

A 10-min video-recorded structured play task was used to assess parent–child interaction quality. Mothers and children completed the Etch-A-Sketch task (Stevenson-Hinde & Shouldice, 1995), and fathers and children completed the Co-Construction Task (Steele et al., 2005). The Etch-A-Sketch is a drawing tool with two dials that allow one participant to draw horizontal lines, and the other to draw vertical lines. Mothers and children were asked to copy a picture of a house using only one dial each. In the Co-Construction task, fathers and children were given a set of wooden building blocks and were asked to build something together, using as many blocks as possible. Interactions were later coded using the fourth edition of the Emotional Availability (EA) Scales (Biringen, 2008). EA is a founded in attachment theory and reflects the dyad’s capacity to share an emotionally healthy relationship. The EA Scales have been found to consistently predict attachment categories and are reliable and valid across contexts (Biringen et al., 2014). VJ and JL were trained in the administration and coding of the EA Scales by the creator of the scales and coded the interviews. The researchers were all experienced in the administration of the Etch-A-Sketch and Co-Construction tasks.

The EA coding scheme measures the affect and behavior of the parent and child. The scheme includes six dimensions (four parent, two child), with each dimension comprising seven items. Each dimension is comprised of two items that are coded from 1 *nonoptimal* to 7 *optimal,* and five items that are coded from 1 *nonoptimal* to 3 *optimal*. Scores for all items on each dimension are summed, to produce a total score for each dimension, with higher scores reflecting more optimal functioning. The four parent dimensions were as follows: *Sensitivity, Structuring, Nonintrusivness,* and *Nonhostility*. *Sensitivity* assesses emotional sensitivity and behavioral sensitivity, specifically examining the appropriateness of the parent’s affect, flexibility of attention and behavior, appropriateness of responding to the child’s signals, attunement to timing, parental acceptance of the child, and ability to resolve conflicts. *Structuring* focuses on the parent’s ability to appropriately guide the child, support their learning, and scaffold their activities so as to involve the child in sustained interactions. *Nonintrusiveness* assesses the parent’s ability to follow the child’s lead without overdirecting, overstimulating or interfering. *Nonhostility* measures the parent’s ability to control their negative emotions and refrain from expressing overt hostility, such as negative statements, or covert hostility, such as boredom, to the child. The two child dimensions were *Child responsiveness to the parent* and *Child involvement of the parent.* The former measures the child’s emotional and behavioral responses to the parent and includes the child’s affect, age-appropriate autonomy seeking, responsiveness to the parent, avoidance and absence of overresponsiveness. The latter assesses the child’s ability to involve the parent, including their attempts to initiate interaction, and any evasiveness displayed in their gaze, body language, or a lack of engagement. One-third of randomly selected mothers’ videos were coded by a second rater to establish interrater reliability. The intraclass correlations for *sensitivity, structuring, nonintrusiveness, nonhostility, child responsiveness,* and *child involvement* for mothers (*N* = 39) were .82, .87, .91, .82, .94, and .72, respectively, and for fathers (*N* = 29) were .92, .88, .91, .88, .92, and .82, respectively.

##### Representational Measure: Parent Development Interview

Parents were administered an adaptation of the Parent Development Interview (PDI; Aber et al., 1985; Henderson et al., 2007), with mothers and fathers interviewed separately. The PDI is derived from attachment theory and is a semistructured interview examining parents’ representations of the parent–child relationship. Parents are asked to describe themselves and their child in moments of relatedness and interaction. PDIs were audio-recorded, transcribed verbatim, and coded using a coding scheme developed by Henderson et al. (2007). The scheme yields codes assessing the parent’s representation of themself as a parent (parent affective experience codes), the parent’s representation of the child (child affective experience codes), and reflective functioning. SI and VJ were trained in the administration and coding of the PDI at the Center for Attachment Research at the New School for Social Research. SI and VJ coded the interviews, ensuring that they did not code mothers and fathers from the same family. They were largely unaware of family type, although occasionally a parent referred to their method of conception. The interviewers (JL, KS, and JG) were trained in the administration of the PDI by SI and VJ.

The parent affective experience codes were each rated on a 4-point scale, with a higher score representing a higher level of the construct: (a) *degree of anger*, assessing the extent to which the parent feels angry in the relationship; (b) *expression of anger*, assessing the degree to which anger is expressed in the relationship; (c) *need for support*, measuring the parent’s acknowledgement of need for support; (d) *satisfaction with available support*, measuring satisfaction with the support available to them; (e) *guilt*, assessing the extent to which guilt is present in the relationship; (f) *joy/pleasure*, measuring the parent’s ability to express feelings of joy in the relationship to and with the child; (g) *competence*, assessing how well the parent is coping with the child; (h) *confidence*, measuring the parent’s sense of their own competence; (i) *level of child focus*, assessing the extent to which the parent is focused on the needs of the child as compared to their own emotional needs; (j) *disappointment/despair*, measuring the degree to which the parent expresses disappointment with being a parent; (k) *warmth*, assessing the amount of warmth the parent feels toward the child; (l) *attachment awareness and promotion*, measuring the parent’s understanding of the attachment issues for their child and their ability to behave in ways that will promote the child’s attachment to them; and (m) *hostility*, assessing hostile feelings toward the child.

The child affective experience codes, used to assess the parent’s representation of the child, were also rated on a 4-point scale, with a higher score representing a higher level of the construct (a) *child anger*, measuring the extent to which the parent represents the child as experiencing/expressing anger; (b) *child happiness*, assessing the degree to which the parent represents the child as happy and contented as distinct from the parent–child relationship; (c) *child controlling/manipulating*, measuring the extent to which the parent represents the child as attempting to control the parent and their interactions; (d) *child affection*, assessing the degree to which the child shows and accepts physical affection in relation to the parent; (e) *child rejection*, measuring the degree to which the parent feels rejected by the child either emotionally or practically. The global code *parental reflective functioning* measures the degree to which the parent can reflect on the child and their relationship. The code captures the extent to which parents are able to “look underneath” the child’s behaviors for explanations, the extent to which they try to understand the child’s behaviors in terms of the child’s early experiences, and the extent to which they consider and evaluate their own contribution to any difficulties. One-third (*N* = 39) of randomly selected mothers’ PDI transcripts were coded by a second rater to establish interrater reliability. Where discrepancies occurred, scores were discussed, and a final code agreed. Intraclass correlations ranged from .70 to .98.

##### Criticism

Parents were administered a semistructured interview designed to assess parenting quality (Quinton & Rutter, 1988). In the interview, parents were asked to talk about detailed accounts of their child’s behavior and the parent’s response to it. Parental criticism was then rated using a standardized coding scheme based on a detailed coding manual. The degree of the parent’s criticism of the child was rated from 1 (*no criticism*) to 5 (*considerable criticism*). One-third of interviews were rated by a second coder. The intraclass correlation was .84.

#### Child Psychological Adjustment

##### SDQ

The SDQ (R. Goodman, 1997) was administered to mothers and teachers to assess child psychological adjustment. The SDQ is a 25-item behavioral screening questionnaire (comprising 5 scales: emotional symptoms, conduct problems, hyperactivity/inattention, peer relationship problems, prosocial behavior), with total scores ranging from 0 to 40, and higher scores indicating greater adjustment problems. The conduct problems and hyperactivity scales were summed to give an “externalizing problems” score of 0 to 20, and the emotional and peer problems scales were summed to give an “internalizing problems” score of 0 to 20 (A. Goodman et al., 2010). The SDQ has strong psychometric properties (Stone et al., 2010). Cronbach’s α for the mother sample = .73, father sample = .70, and for the teacher sample = .85.

##### Ratings of Psychiatric Disorder

A section of the interview with the mother was used to assess the presence of child psychiatric disorder using a standardized procedure (Rutter et al., 1975). Mothers were asked to provide a detailed description of any emotional or behavioral problems displayed by the child, and information was gathered about the frequency, severity, precipitants, and course of behaviors over the last year. This was transcribed verbatim and rated by a child psychiatrist who was unaware of the child’s family background. A high level of agreement has been demonstrated between mothers’ assessments of their children’s emotional/behavioral difficulties and interview ratings (Rutter et al., 1975). Ratings were made on a 4-point scale: 0 = *none*, 1 = *slight*, 2 = *definite*, 3 = *marked*. Type of disorder was identified as: emotional, conduct, mixed, developmental, attention deficit hyperactivity disorder, psychotic, or other.

### Analysis Plan

To address the hypotheses regarding differences between family types in the mother, father, and child measures at age 5, univariate and multivariate analyses of variance were used and demographic covariates included when they differed between family types and were associated with the outcome measure. Parental age was not controlled for as it is a defining characteristic of the groups, with egg donation parents known to be older parents (Golombok et al., 2005).

Given the longitudinal nature of the study, we used latent change score (LCS) models to examine intraindividual change over time in predictors (McArdle, 2009) and used these scores to explore predictors of child adjustment problems. Where possible, our outcome measures reflected a composite score of maternal and paternal SDQ ratings. First, LCS models were used to examine intraindividual change over time in maternal measures (McArdle, 2009). For example, the LCS model can be expressed as:

Phase 2 Reflective Functioning=1 Phase 1 RF+1(Phase 2 RF−Phase 1 RF). 1

This first involves fixing the regression weight of the score at Time 2 as a function of Time 1 to 1. Then a latent factor score (e.g., ΔReflective Functioning) is defined by subtracting the Time 1 score from Time 2 with a factor loading fixed to 1. This interindividual change in the latent factor score can be examined error free and can subsequently be used as a predictor or outcome of interest. Thus, building on this initial model, Phase 2 child externalizing and internalizing scores were then regressed onto the LCSs, as well as the Phase 2 specific parenting measures (maternal criticism and observed mother–child interaction quality). Finally, to test whether family type moderated any of the associations, interaction terms were created. Specifically, predictor variables were first centered within Mplus and subsequently multiplied with family type, and then entered into the model. All of the models were run using Mplus Version 8 (Muthén & Muthén, 2012) and model fit was evaluated using Brown’s (2015) recommended criteria: nonsignificant χ^2^, root-mean-square error of approximation (RMSEA) < .08, comparative fit index (CFI) > .90, and Tucker–Lewis index (TLI) > .90. As highlighted above, our LCS models were carried out using maternal measures as only 9.2% of mothers did not complete some of the Phase 2 questionnaire measures. Thus, we used a full information approach so that all eligible families were included in the model (*N* = 122 families). This sample size gave us 80% power to detect medium size effects. Unfortunately, the proportion of missing data for fathers interview and questionnaire measures exceeded the widely established ≥ 20% threshold which would compromise any inferences (i.e., 29% missing paternal interview data and 20% questionnaire data). The sample size gave us 80% power to detect medium size effects. The data are not publicly accessible due to the potentially identifiable nature of the sample.

## Results

### Comparisons of Well-Being and Parenting by Family Type

Descriptive statistics and group differences by family type for the key Phase 2 measures are presented in Table 2.[Table tbl2]

#### Mothers

Phase 2 measures of parenting stress, couple relationship quality, social support, and criticism were entered into a multivariate analysis of variance (MANOVA), with maternal education included as a covariate. There were moderate significant differences in parenting stress between family types, with mothers in egg donation families reporting significantly higher levels of stress compared to mothers in IVF families, *F*(1, 88) = 5.30, *p* = .024, Cohen’s *d* = 0.48. Similarly, there were moderate significant differences between family types in social support and relationship quality, with mothers in egg donation families reporting lower perceived levels of support compared with IVF mothers, *F*(1, 88) = 5.70, *p* = .019, Cohen’s *d* = 0.50, and poorer relationship quality than IVF mothers, *F*(1, 88) = 5.67, *p =* .019, Cohen’s *d* = 0.49. There was no significant difference between family types in criticism.

#### Fathers

Phase 2 measures of parenting stress, couple relationship quality, social support, and criticism were entered into a MANOVA, with paternal education included as a covariate. There were moderate significant differences between family types in parenting stress, with fathers in egg donation families reporting higher levels of stress compared to IVF fathers, *F*(1, 77) = 5.75, *p* = .019, Cohen’s *d* = 0.55. There were also moderate significant differences between family types in criticism, with egg donation fathers expressing higher criticism of their children than fathers in IVF families, *F*(1,77) = 4.80, *p* = .032, Cohen’s *d* = 0.50. There were no significant differences between family types in couple relationship quality or perceived social support.

### Comparisons of Parent–Child Relationship Quality (Observational Measure) by Family Type

#### Mothers

Phase 2 observational variables of mothers’ sensitivity, structuring, nonintrusiveness, nonhostility and child responsiveness, and child involvement were entered into a MANOVA, with maternal education, child age, and sex included as covariates. As illustrated in Table 2, there were no significant differences between family types in either the maternal or child observational measures.

Following this, confirmatory factor analysis was conducted on the observed mother–child interaction quality variables. A one-factor solution reflecting dyadic interaction quality was tested and provided an excellent fit to the data, χ^2^(5) = 1.46, *p* = .918, RMSEA = .000, 90%CI [.00, .05], CFI = 1.00, TLI = 1.00. There was no significant difference in the quality of observed dyadic interaction between egg donation and IVF families, Cohen’s *d* = 0.23. This factor score was used in subsequent analyses.

#### Fathers

Observational measures of fathers’ sensitivity, structuring, nonintrusiveness, nonhostility, child responsiveness, and child involvement were entered into a MANOVA, with paternal education, child age, and sex included as covariates. There was a significant difference between family types in structuring, with egg donation fathers rated lower on structuring than fathers in IVF families, *F*(1, 82) = 5.17, *p* = .026, Cohen’s *d* = 0.52. There were no significant differences between groups in any of the other paternal or child observational scales (see Table 3).[Table tbl3]

### Comparisons of Parent–Child Relationship Quality (Representational Measure) by Family Type

#### Mothers

MANOVAs controlling for maternal education found significant differences on four mother and two child variables from the Parent Development Interview. Mothers in egg donation families represented themselves as significantly higher in degree and expression of anger and as significantly less confident and competent than IVF mothers and represented their children as higher in anger and lower in happiness than did IVF mothers (see Table 2). There were no significant differences between family types on any of the other maternal PDI variables (see Table 2).

#### Fathers

MANOVAs controlling for paternal education found significant differences between groups for five father and one child variables. Egg donation fathers represented themselves as significantly higher in expression of anger, and significantly lower in satisfaction with support, joy, confidence, and competence than IVF fathers (see Table 3). There were no significant differences between family types on any of the other paternal PDI variables (see Table 3).

### Comparisons of Child Adjustment by Family Type

As shown in Table 2, a MANOVA controlling for child age and sex found a moderate significant difference between family types for mother-reported externalizing problems, such that children in egg donation families were reported to have higher problems compared to IVF families, *F*(1, 111) = 6.13, *p* = .015, Cohen’s *d* = 0.48. However, there were no differences between families in children’s internalizing problems, Cohen’s *d* = 0.10. Similar elevated results in egg donation compared with IVF families were found for father-reported externalizing problems, *F*(1, 89) = 8.05, *p* = .006, Cohen’s *d* = 0.60. No differences were found between families in fathers’ reports of children’s internalizing problems, Cohen’s *d* = 0.11.

Turning to teacher-reported child adjustment problems, a MANOVA controlling for child age and sex, found a moderate significant difference between family types for teacher-reported externalizing problems, such that children in egg donation families were reported to have higher levels of problems compared to IVF families, *F*(1, 77) = 4.23, *p* = .043, Cohen’s *d* = 0.47. There was also a moderate significant difference between family types for teacher-reported internalizing problems, such that egg donation children were reported to have higher problems compared to IVF children, *F*(1, 77) = 5.69, *p* = .020, Cohen’s *d* = 0.53.

Regarding the child psychiatrist’s ratings, the severity of psychiatric problems did not differ between groups (i.e., no disorder, slight, definite, or marked) between the egg donation and IVF families, χ^2^(3) = 1.12, *p* = .77. For the entire sample, 10.7% (*n* = 13) of the children were rated as having a psychiatric problem, of whom three showed emotional problems, three were rated as having conduct problems, two were rated as having attention deficit hyperactivity disorder and five had developmental or mixed developmental and behavioral problems.

### Changes in Family Processes Over Time and Children’s Adjustment at Age 5

Unstandardized results from the just-identified LCS model indicated that maternal parenting stress and maternal couple relationship dissatisfaction significantly increased across early childhood, Mean ΔPSI = 7.71, 95% CI [4.91, 10.51], *p* < .0001, and, Mean ΔGRIMS = 3.03, 95% CI [1.69, 4.34], *p* < .0001, respectively. The variance in each of the LCSs differed significantly from 0, maternal reflective functioning, parenting stress, social support, and couple relationship quality, *p* > .0001. The rate of change in all measures (aside from parenting stress) across early childhood was stronger for those with lower initial levels; maternal reflective functioning, *r* = −.44, *p* < .0001, social support, *r* = −.48, *p* < .0001, and couple relationship dissatisfaction, *r* = −.23, *p* < .0001.

Following this, child externalizing and internalizing problem scores were regressed onto latent scores reflecting the initial level and the LCSs for maternal reflective functioning, parenting stress, social support, and couple relationship quality across early childhood (i.e., 1 year to 5 years), and concurrent maternal criticism, dyadic interaction quality, child age, child sex, and family type were also included. This model showed a good fit to the data, χ^2^(14) = 15.22, *p* = .363, RMSEA = .022, 90%CI [.00, .08], CFI = 0.983, TLI = 0.967. As illustrated in Table 4, over and above the effects of child age and sex, lower initial levels of social support, fewer changes in reflective functioning, steeper increases in parenting stress and greater concurrent maternal criticism were associated with elevated externalizing scores at age 5. On the other hand, poorer initial couple relationship quality and fewer gains in reflective functioning across early childhood were associated with higher internalizing scores at age 5.[Table tbl4]

Finally, although family type did not exert a significant main effect, interaction terms between predictor variables and family type were created and added into the model to test whether family type moderated any of the associations. There was an interaction between the parenting stress LCS and family type, Est = .08, *p* = .003, showing that steeper increases in parenting stress over time were associated with higher levels of child externalizing problems for egg donation, but not IVF, families. All other interactions were nonsignificant.

## Discussion

This study examined the quality of parent–child relationships, parental psychological well-being, and children’s adjustment in families with 5 year olds conceived by identity-release egg donation, in comparison with families created by IVF with the parents’ own gametes. There were no differences between family types in mother–child or father–child interaction quality, apart from lower structuring by fathers in egg donation families. However, mothers and fathers in egg donation families showed higher levels of parenting stress and represented themselves as less confident and competent as parents, than IVF mothers and fathers. Egg donation mothers reported lower levels of social support and couple relationship quality, greater anger toward their child, and perceived their child as more angry and less happy, compared to IVF mothers, whereas egg donation fathers showed greater criticism and anger toward their child, less joy in parenting, and were less satisfied with the support they received, than IVF fathers. Children in egg donation families showed higher levels of externalizing problems than IVF children as rated by mothers, fathers, and teachers, whereas they were rated as having higher levels of internalizing problems by teachers only.

At Phase 1 of the study, mothers and infants in egg donation families had shown less optimal interaction quality (Imrie, Jadva, Fishel, & Golombok, 2019), a difference that may be explained by some mothers in egg donation families finding it challenging to adjust to nongenetic parenthood, perhaps because of a lack of physical resemblance with their child. In the present phase of the study, some mothers reported that knowing what the donor looked like might interfere their relationship with their child (Lysons et al., 2022, 2023). However, no differences were identified between family types on the observational measure of interaction quality. This finding is in contrast to those from the U.K. Longitudinal Study of Assisted Reproduction families when the children were aged seven, in which less positive mother–child interaction was found in egg donation families compared to sperm donation families (Golombok et al., 2011). Whereas the authors of this previous study suggested that this could be explained by families’ disclosure status, it was not possible to explore this variable in the present study as the majority of parents intended to disclose the donor conception to their child. The absence of differences in mother–child interaction is, however, consistent with children’s ratings of mother–child relationship quality, assessed using the Berkeley Puppet Interview in the current sample (Imrie et al., 2021), which found that children in egg donation families rated their mothers as higher in warmth/enjoyment of the mother–child relationship than did children in IVF families. The finding that egg donation fathers were less structuring in their play than were IVF fathers was not found at Phase 1 and also contrasts with findings from fathers with 7 year olds conceived through anonymous egg donation (Casey et al., 2013).

In terms of parental representations of the parent–child relationship, more similarities than differences were found between family types, as was the case in the first phase of the study (Imrie, Jadva, Fishel, & Golombok, 2019), and in the only other study to have compared genetically related and unrelated mother–child dyads in families formed by egg donation (Golombok et al., 2005). Where differences were identified between family types, they indicated less positive representations among the egg donation than the IVF parents. However, the mean scores on all six of the Emotional Availability scales for mothers and fathers were in the upper quarter of the scale, indicating good parent–child relationship quality in both family types. That parents and children in both family types scored highly for Emotional Availability suggests probable positive future developmental outcomes for children, as Emotional Availability is predictive of attachment categories (Easterbrooks & Biringen, 2000), and in samples of preschool children has been found to be associated with a range of positive outcomes, including school readiness (Biringen et al., 2005) and social competence (Howes & Hong, 2008).

As parental caregiving representations are known to be associated with observed parental behaviors, and with child attachment classifications (George & Solomon, 1996), understanding the parent–child relationship at both the representational level and behavioral level is important for understanding the organization of the relationship from an attachment perspective (Korja et al., 2010). Both measures in the present study indicate a high quality of parent–child relationship in both family types.

That parents in egg donation families had poorer scores on several measures of psychological well-being, namely higher parenting stress for both mothers and fathers, and lower perceived social support and poorer relationship quality than IVF parents in the case of mothers, is to some extent consistent with Phase 1 of the study, in which egg donation mothers similarly reported lower social support during infancy (Imrie et al., 2019). It is conceivable that perceptions of lower levels of social support may be related to egg donation mothers’ older age. Maternal age has been identified as a factor associated with lower social support in samples of mothers with preschool children born through assisted reproduction (Mac Dougall et al., 2012). It may be that the family and friends of older parents, being older themselves, may be less able to provide adequate support, and future research with this family type that distinguishes between different types of support (e.g., practical, emotional, financial) may be beneficial. Parenting stress was not found to be higher among egg donation families compared to IVF families in the first phase of the study (Imrie et al., 2019) but was associated with age in analyses examining the effects of age among egg donation parents in the current sample (Jadva et al., 2022). The physical demands associated with parenting may become more challenging as parents age (Meyer, 2020), and older parents may experience increased judgment from other parents (Jadva et al., 2022).

In terms of child psychological adjustment, children in egg donation families were found to have higher levels of externalizing problems as rated by mothers, fathers, and teachers, and higher levels of internalizing problems as rated by teachers. However, mean scores for both groups for externalizing and internalizing problems were in the normal range and indicate that both groups of children had good psychological adjustment. This is in line with findings from the two British studies of anonymous egg donation families (Golombok et al., 2013; Shelton et al., 2009), and a Swedish study of identity-release egg donation families that also used the SDQ and found children’s scores to be within the normal range (Widbom et al., 2022).

Latent change score modeling, conducted to examine predictors of children’s adjustment, revealed that elevated child externalizing scores were predicted by mothers’ lower initial levels of social support, fewer changes in maternal reflective functioning, steeper increases in parenting stress across early childhood and greater concurrent maternal criticism. Poorer initial couple relationship quality and fewer changes in maternal reflective functioning across early childhood were associated with higher internalizing scores. These findings are in line with a relational developmental systems approach (Osher et al., 2020; Overton, 2015). They are also consistent with the bodies of literature that have identified reduced social support, parenting stress, and critical parenting as risk factors for child externalizing problems (Neece et al., 2012), and longitudinal studies that have found associations between couple relationship quality and internalizing problems in toddlerhood (Hughes et al., 2020) and middle childhood (Brock & Kochanska, 2016). Family type did not exert a significant main effect, indicating that these risk factors were functioning in a similar manner in both egg donation and IVF families. There was, however, an interaction such that steeper increases in parenting stress over time were associated with higher child externalizing problems at age five for egg donation, but not IVF, families, suggesting that the effects of increased parenting stress in earlier childhood may be more problematic for egg donation families.

That fewer changes in maternal reflective functioning across early childhood were associated with both higher externalizing and internalizing scores is in line with attachment theory and contributes to the literature on the role of parental reflective functioning in child development, which has primarily focused on the relations between parental reflective functioning and child attachment security and child mentalization (Luyten et al., 2020). Associations have also been found between maternal reflective functioning and child adjustment in a low-risk sample of Iranian families (Khoshroo & Seyed Mousavi, 2021), and between parental reflective functioning and parent–child interaction in adoptive families (Leon et al., 2018). However, the present study is the first to identify a link between *fewer gains* in reflective functioning over early childhood and child adjustment problems, thus increasing understanding of the correlates and consequences of differences in parental reflective functioning. Higher reflective functioning enables parents to try to identify the reasons behind problem behaviors and engage constructively with their child (Khoshroo & Seyed Mousavi, 2021), and so it is perhaps not surprising that child adjustment difficulties in the current sample were higher in families in which mothers’ reflective functioning did not increase in the early years as it suggests that they may have found it challenging to alter their representations as their child developed. Interventions aimed at increasing parental reflective functioning during infancy and toddlerhood have been found to show improvements (Barlow et al., 2021) so may be a useful target for focusing support.

A limitation of the current investigation is the homogeneity of the sample, which was predominantly comprised of highly educated parents who identified their ethnicity as White British. While this is representative of the families who are typically able to access IVF treatment with egg donation in the United Kingdom, the majority of which is privately funded (Human Fertilisation and Embryology Authority, 2021), it does limit the extent to which the findings can be generalized to other sociocultural contexts. Similarly, as all families were heterosexual, cisgender two-parent families, the findings cannot be generalized to parents in other family structures who may use egg donation in their path to parenthood (e.g., families using egg donation in a surrogacy arrangement, single women using donor sperm and eggs).

Over 80% of families remained in the study at Phase 2. While this can be considered a high retention rate for a longitudinal study (Abshire et al., 2017), and the retained sample did not differ from those who did not participate in key demographic variables or in the main Phase 1 variables of interest, it cannot be ruled out that parents who were experiencing greater difficulties with their child, or who felt less comfortable discussing their child’s method of conception, may have been less inclined to participate at follow-up. Parents have cited not wanting to be reminded of the nongenetic relationship with their child as a reason for nonparticipation in the previous studies of reproductive donation families (Golombok et al., 1995). In addition, while the overall retention rate can be considered high, fewer fathers took part in both waves. Therefore, sample size restraints precluded our ability to examine the unique and additive effects of fathers’ measures on child adjustment problems. We look forward to other researchers taking these findings forward and testing this model on a larger and more diverse sample.

Nevertheless, the present study offers the first longitudinal research into family functioning in families formed by identity-release egg donation, a method of conception that is increasing sharply in use (Centers for Disease Control and Prevention, 2021; Human Fertilisation and Embryology Authority, 2020) and will continue to do so as growing numbers of countries prohibit the use of anonymous donation (Calhaz-Jorge et al., 2020), making this investigation particularly timely. The sample size remains the largest to date in studies of families created using egg donation that use in-depth interview and observational measures to assess family functioning and a multi-informant design. Given the challenges involved in recruiting reproductive donation families to research on topics associated with perceived or real stigma (Nachtigall et al., 1997), the current sample can be considered relatively large.

The findings of the study showed that egg donation using an identifiable donor contributed to greater challenges than IVF using the parents’ own gametes. Therapeutic support, such as 1:1 counseling sessions, whether postconception or postbirth, would provide parents with an opportunity to explore their feelings about, and issues surrounding, their use of identifiable egg donation in a safe and structured way. Group workshops may similarly prove an effective means for disseminating information to parents of children born through egg donation, including additional details about donation type that parents may have been unable or unwilling to engage with at the treatment stage. Workshops may also provide parents who have used identity-release donation with the chance to meet with other donor conception parents, thereby facilitating discourse in which their shared experiences, both positive and negative, may be expressed, normalized, and legitimized.

Overall, the egg donation families were more similar than different to the IVF families, and scores on all measures of parent–child relationship quality and child psychological adjustment were within the normal range. This should prove reassuring to existing families created using egg donation, clinicians, and prospective parents considering their treatment options. That significant differences were found between family types in maternal psychological well-being, and that psychological well-being variables were identified as associated with increased levels of adjustment problems for children, suggest that some assisted reproduction families may benefit from additional psychological support beyond their child’s first year of life.

## Figures and Tables

**Table 1 tbl1:** Sociodemographic Information by Family Type

Variable	IVF (*n* = 50)		Egg donation (*n* = 72)		*t*	*p*
	*M*	*SD*	*M*	*SD*		
Age of mother (years)	42.28	3.93	47.63	4.35	6.94	.000
Age of father (years)	44.29	8.41	48.48	6.48	3.11	.000
Age of child (years)	5.63	.32	5.63	.32	.08	.938

	*n*		*n*		χ^2^	*p*
Child gender					.52	.471
Male	29		37			
Female	21		35			
Siblings						
None	13		38		9.65	.008
1	26		27			
2+	9		5			
Mother’s working status					1.86	.601
Not working	5		10			
Part-time	27		36			
Full-time	16		22			
Other	0		2			
Father’s working status					4.08	.253
Not working	1		0			
Part-time	4		10			
Full-time	37		57			
Other	0		3			
Perceived financial difficulties					1.18	.555
None	41		63			
Minor	6		6			
Definite	0		1			
Mother’s education					.69	.405
Below university degree	19		22			
Undergraduate degree+	26		42			
Father’s education					.01	.940
Below university degree	20		30			
Undergraduate degree	22		34			
Mother’s psychiatric contact					.89	.829
None	34		54			
General practitioner	12		13			
Outpatient	1		1			
Father’s psychiatric contact					2.33	.127
None	30		50			
General practitioner	0		4			
Outpatient	0		0			
*Note*. IVF = in vitro fertilization.						

**Table 2 tbl2:** Mother and Child Adjustment and Relationship Quality by Family Type

Phase 2 measure	IVF		Egg donation		*F*	*p*	Cohen’s *d*
	*M*	*SD*	*M*	*SD*			
Externalizing problems^a^							
Mother report	4.62	2.64	6.07	3.17	6.13	.015	0.48
Teacher report	1.69	2.30	3.11	3.52	4.23	.043	0.47
Internalizing problems^a^							
Mother report	2.33	2.10	2.67	2.31	.29	.590	0.10
Teacher report	2.40	3.00	4.28	3.89	5.69	.020	0.53
Psychological well-being^a^							
Parenting Stress Index	61.57	15.94	71.12	22.35	5.30	.024	0.48
Social support	6.18	.72	5.64	1.27	5.70	.019	0.50
Couple relationship quality	22.72	11.62	28.38	11.29	5.67	.019	0.49
Mother–child interaction quality^b^							
Mother sensitivity	24.63	2.80	24.56	2.92	.02	.883	0.02
Mother structuring	24.81	2.65	24.53	3.21	.12	.726	0.09
Mother nonintrusiveness	22.23	3.77	23.13	3.52	.95	.331	0.25
Mother nonhostility	26.22	2.36	26.35	2.43	.00	.974	0.05
Child responsiveness	24.50	2.65	24.20	3.27	.41	.522	0.10
Child involvement	23.87	2.12	23.56	3.48	.35	.558	0.10
Parenting quality interview^c^							
Mother criticism	1.11	.73	1.23	.67	.45	.504	0.17
Maternal representations^d^							
Reflective functioning	3.29	.70	3.25	.58	.37	.543	0.13
Degree of anger	2.21	.47	2.53	.42	14.37	.000	0.80
Expression of anger	1.98	.70	2.26	.66	4.85	.030	0.47
Need for support	2.02	.48	1.91	.53	1.30	.256	0.24
Satisfaction with support	3.71	.64	3.71	.52	.01	.913	0.02
Guilt	2.26	.62	2.16	.72	.53	.468	0.15
Joy/pleasure	3.40	.55	3.31	.56	.60	.441	0.16
Competence	3.44	.55	3.32	.58	4.36	.039	0.44
Confidence	3.37	.56	3.13	.60	4.27	.041	0.44
Child focus	3.55	.53	3.46	.56	.97	.328	0.21
Disappointment/despair	1.36	.53	1.52	.58	1.63	.204	0.27
Warmth	3.73	.50	3.69	.53	.29	.595	0.11
Attachment awareness	3.51	.57	3.37	.58	2.38	.126	0.33
Hostility	1.07	.21	1.17	.43	2.65	.107	0.35
Child anger	1.98	.70	2.37	.83	6.28	.014	0.53
Child happiness	3.37	.51	3.18	.48	4.09	.046	0.43
Child controlling	1.72	.57	1.95	.62	3.71	.057	0.41
Child affection	3.71	.45	3.61	.55	.72	.400	0.20
Child rejection	1.29	.40	1.45	.51	2.80	.097	0.35
*Note*. IVF = in vitro fertilization.							
^a^ Questionnaire. ^b^ Observation. ^c^ Semistructured interview global code. ^d^ Parent Development Interview.							

**Table 3 tbl3:** Father and Child Adjustment and Relationship Quality by Family Type

Phase 2 measure	IVF		Egg donation		*F*	*p*	Cohen’s *d*
	*M*	*SD*	*M*	*SD*			
Child adjustment (father report)^a^							
Externalizing problems	4.69	2.59	6.56	3.99	8.05	.006	0.60
Internalizing problems	2.77	3.01	3.22	2.99	.27	.602	0.11
Psychological well-being^a^							
Parenting Stress Index	56.86	13.06	66.81	18.38	5.75	.019	0.55
Social support	5.90	.86	5.65	.998	1.08	.302	0.24
Couple relationship quality	19.31	11.75	22.19	10.47	.81	.372	0.21
Father–child interaction quality^b^							
Father sensitivity	25.00	3.27	23.71	3.73	3.12	.081	0.41
Father structuring	24.14	4.56	21.98	4.35	5.17	.026	0.52
Father nonintrusiveness	24.22	3.70	23.74	3.38	.65	.422	0.19
Father nonhostility	26.69	2.34	26.04	3.43	.90	.346	0.22
Child responsiveness	25.00	3.72	23.99	3.76	1.50	.224	0.28
Child involvement	24.64	3.71	23.94	4.35	.35	.558	0.14
Parenting quality interview^c^							
Father criticism	.79	.68	1.17	.73	4.80	.032	0.50
Paternal representations^d^							
Reflective functioning	3.07	.77	2.92	.64	1.19	.279	0.25
Degree of anger	2.05	.49	2.24	.57	2.25	.138	0.35
Expression of anger	1.73	.63	2.08	.77	4.36	.040	0.48
Need for support	1.36	.43	1.60	.66	2.51	.117	0.36
Satisfaction with support	3.97	.18	3.68	.65	4.94	.029	0.51
Guilt	1.77	.51	1.88	.60	.60	.442	0.18
Joy/pleasure	3.45	.62	3.13	.64	4.81	.031	0.50
Competence	3.47	.46	3.04	.66	10.72	.002	0.75
Confidence	3.50	.41	3.04	.66	8.50	.005	0.67
Child focus	3.44	.51	3.29	.59	1.64	.204	0.29
Disappointment/despair	1.23	.44	1.39	.52	1.51	.223	0.28
Warmth	3.53	.60	3.38	.66	1.02	.317	0.23
Attachment awareness	3.34	.57	3.11	.59	2.85	.095	0.39
Hostility	1.05	.20	1.14	.31	1.50	.225	0.28
Child anger	1.86	.71	2.03	.66	.85	.359	0.21
Child happiness	3.36	.52	3.26	.52	.65	.422	0.19
Child controlling	1.63	.61	1.92	.64	3.86	.053	0.45
Child affection	3.58	.55	3.52	.65	.11	.736	0.08
Child rejection	1.24	.48	1.39	.56	1.61	.208	0.29
*Note*. IVF = in vitro fertilization.							
^a^ Questionnaire. ^b^ Observation. ^c^ Semistructured interview global code. ^d^ Parent Development Interview.							

**Table 4 tbl4:** Unstandardized and Standardized Estimates for Correlates of Child Externalizing and Internalizing Problems at Age 5

Variable	Externalizing problems			Internalizing problems		
	Est.	*SE*	β	Est.	*SE*	β
Demographics						
Family type	.81	.47	.14	.00	.39	.00
Child age	2.20	.68	.24**	−.07	.64	−.01
Child sex	.59	.43	−.16*	.59	.43	.13
Phase 1: 12 months						
Maternal reflective functioning	−.45	.42	−.09	−.76	.47	−.20
Maternal parenting stress	.02	.02	.09	.01	.02	.06
Maternal social support	−.59	.25	−.21*	−.02	.25	−.01
Couple relationship quality	−.01	.03	−.04	.06	.02	.25*
Latent change scores						
Δ Reflective functioning	−.93	.37	−.18*	−.79	.42	−.21^†^
Δ Parenting stress	.04	.02	.22*	.01	.02	.10
Δ Maternal social support	.27	.20	.11	.29	.17	.15
Δ Couple relationship quality	.04	.03	.10	.02	.02	.08
Phase 2: 5 years						
Observed dyadic interaction quality	−.17	.10	−.15^†^	−.03	.09	−.04
Maternal criticism	.91	.33	.23**	.07	.32	.02
*R* ^2^			.40			.17
*Note*. Δ = latent change scores; family type (egg donation = 1). Est. = estimated; *SE* = standard error.						
* *p* < .01. ** *p* < .01. ^†^ *p* < .10.						
